# An Experimental and Clinical Study of Flap Monitoring with an Analysis of the Clinical Course of the Flap Using an Infrared Thermal Camera

**DOI:** 10.3390/bioengineering11070688

**Published:** 2024-07-07

**Authors:** Hyun Kim, Si Hyun Kwak, Je Yeon Byeon, Da Woon Lee, Jun Hyuk Kim, Soomin Lim, Hwan Jun Choi

**Affiliations:** 1 Department of Plastic and Reconstructive Surgery, Soonchunhyang University Cheonan Hospital, Cheonan 31151, Republic of Korea; 140845@schmc.ac.kr (H.K.); 132513@schmc.ac.kr (S.H.K.); 115954@schmc.ac.kr (J.Y.B.); 103022@schmc.ac.kr (D.W.L.); psdoctor@schmc.ac.kr (J.H.K.); 2 Bachelor of Medicine and Bachelor of Surgery (MBBS), UCL Medical School, University College London, London WC1E 6BT, UK; iamsoominlim@gmail.com

**Keywords:** free-tissue flaps, surgical flaps, differential thermal analysis

## Abstract

Flap surgery is a common method used to cover defects following tumor ablation, trauma, or infection. However, insufficient vascularity in the transferred flap can lead to flap necrosis and failure. Proper postoperative monitoring is essential to prevent these complications. Recently, research has explored the use of infrared thermal imaging in plastic surgery, leading to its clinical application. This study comprises two separate parts: an in vivo experimental study and a clinical study. In this study, 28 rats underwent reverse McFarlane flap surgery, and their flaps were analyzed using a FLIR thermal imaging camera seven days post-surgery. Additionally, thermal images of flaps were taken on postoperative days 0, 1, 2, 3, and 7 in 22 patients. This study focused on temperature differences between normal skin and the perforator compared to the average flap temperature. Results showed that the temperature difference was higher in the necrosis group and increased over time in cases of total necrosis. A lower perforator temperature compared to the flap’s average indicated vascular compromise, potentially leading to flap failure. The FLIR camera, being contact-free and convenient, shows promise for understanding and inferring the clinical progression of flaps in postoperative monitoring.

## 1. Introduction

Flap surgery is a technique in plastic and reconstructive surgery where any type of tissue is lifted from a donor site and moved to a recipient site with an intact blood supply. This is performed to fill a defect such as a wound as a consequence of injuries or surgery when the remaining tissue is unable to support a graft or to rebuild more complex anatomic structures such as the breast or jaw. It stands as a cornerstone of reconstructive procedures, playing a pivotal role in the restoration of form and function following trauma, oncologic resection, or congenital malformation [1]. The success rate of flap surgery relies on meticulous surgical technique and postoperative monitoring to assess tissue viability and mitigate complications [2].

Postoperative monitoring of free flaps is important in flap survival, as immediate action increases the flap salvage rate. Although various methods are available, room for improvement remains. Currently, free flap monitoring can be performed through a variety of methods, including physical examination (skin color, turgor, temperature, and capillary refill), surface temperature recording, external Doppler, implantable Doppler, color duplex sonography, laser Doppler flowmetry, infrared thermography, near-infrared spectroscopy, white light spectroscopy, and microdialysis. Despite the extensive exploration of monitoring methodologies, the absence of a definitive gold standard persists, which necessitates continued investigation into novel approaches [3,4].

Thermal cameras have proven their value in medicine and are currently readily available at a low cost. Their non-invasive nature and ability to provide immediate, quantitative data make infrared thermal imaging an attractive adjunct in flap monitoring [5]. Various clinical applications involving depth analysis of burns, detection of complications for diabetic foot ulcers, and preoperative planning of perforators using infrared thermal imaging have been made in plastic surgery [6,7]. Furthermore, there have been several publications describing flap monitoring using infrared thermal imaging [5].

This study aims to present a new perspective on the clinical course of flaps in flap monitoring by taking advantage of the convenience of forward-looking infrared (FLIR) thermal cameras, focusing on the correlation between flap necrosis and temperature comparison of flaps from a different thermal analysis perspective than previous studies.

## 2. Materials and Methods

### 2.1. Study Design

**Figure 1:**
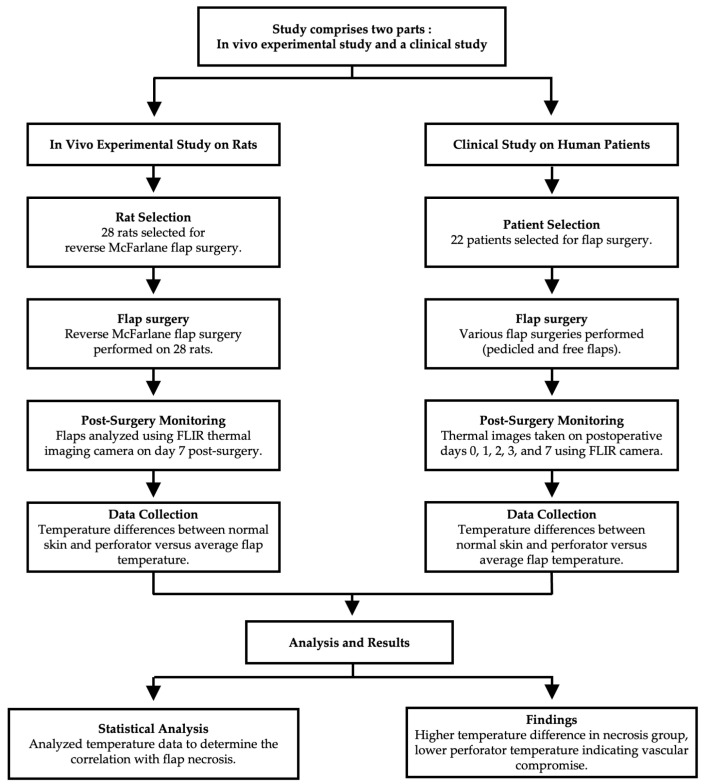
Study design overview. The diagram illustrates the structure and flow of the in vivo experimental study and the clinical study.

This study comprises two main parts: an in vivo experimental study on rats and a clinical study on human patients. The overall study design is illustrated in Figure 1.

### 2.2. Rat Models

**Figure 2:**
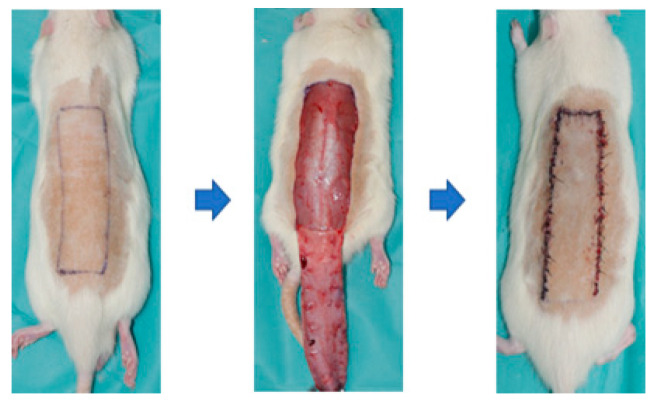
Schematic diagram of surgical procedures. Incisions were made on the back of the mouse under anesthesia with isoflurane, and a 3 × 9 cm sized reverse McFarlane skin flap was elevated using a blade. The flap was immediately closed with sutures using 4-0 nylon.

The in vivo experimental protocols and study were approved by the Institutional Animal Care and Use Committee (IACUC) of Soonchunhyang University (approval number: SCH-0062). In total, 28 transgenic Sprague–Dawley (SD) rats (12 weeks) were involved in this study. On the day of implantation, all rats were trimmed over the backside under anesthesia with isoflurane (Terrel, TX, USA), oxygen, and N_2_O. In this study, a reverse McFarlane skin flap of 3 × 9 cm size was elevated using a blade to induce skin necrosis models to evaluate skin flaps. The flap was immediately closed by suturing with 4-0 nylon (AILEE Co., Seoul, Republic of Korea) (Figure 2). The entire area was covered by gauze and then appropriately bandaged without disturbing the gauze over the wounds. To relieve their pain and prevent infection, tramadol and enrofloxacin were given intramuscularly. All rats were individually isolated in each cage at controlled temperatures (25 °C) and sacrificed on the planned time points (Day 7). The experimental photographs were taken using a Cannon EOS R6 Mark II (Tokyo, Japan) camera at a standard distance (40 cm) to assess the wounds and flaps on postoperative day (POD) 3 and POD 7. The thermal images were obtained by FLIR camera on POD 3 and POD 7. We calculated the ratio of necrotic area/total area and analyzed the difference between necrotic area and non-necrotic area using Fiji-ImageJ software 1.54j (ImageJ, Bethesda, Maryland, USA).

To validate our hypothesis regarding the temperature differences between the necrotic area and the total flap area at POD3 and POD7, we employed three statistical methods: paired *t*-test, regression analysis, and generalized linear mixed models (GLMM) with Gaussian families. The paired *t*-test was used to compare the mean temperature differences between the necrotic area and the total flap area at POD3 and POD7 to determine if there were significant differences between the two time points. Linear regression was performed to assess whether the initial dorsal skin temperature could predict the necrotic area temperature. Finally, Gaussian GLMMs were used to account for the repeated measures within each rat, modeling the necrotic area temperature as a function of pre-flap temperature and total flap temperature at both POD3 and POD7.

### 2.3. Patients

The protocol of this study was approved by the Institutional Review Board (IRB number: 2023-12-071). Patients who underwent pedicled flap or free flap surgery on any locations of the body between December 2021 and September 2023 were included. Patients with distinct hypothermia or hyperthermia were excluded. Also, the buried flaps were excluded. All patients were advised to take absolute bed rest for a week and given intravenous antibiotics for preoperative and postoperative infection control. The clinical photographs were taken using an ILCE-7M4 (Sony^®^, Tokyo, Japan) camera at a standard distance (40 cm) to assess the wounds and flaps.

### 2.4. Infrared Thermal Imaging

Thermal images were captured using the FLIR C5 camera (Teledyne FLIR LLC, Wilsonville, OR, USA). The FLIR C5 has two cameras, a thermal imager (160 × 120 pixels) and 5-megapixel visual camera (640 × 480 pixels), and LED flashlight. Two images were obtained and merged by the Multi-spectral Dynamic Imaging (MSX) technology, resulting in one thermal image with resolution of 640 × 480 pixels. It has the capacity to detect temperatures ranging from -20 °C to 400 °C, and the thermal sensitivity is <70 mK.

All flaps were monitored at POD 0, 1, 2, 3, and 7, and some flaps were even monitored for up to 2 weeks with FLIR camera and clinical findings such as skin color and capillary refill time. The thermal imaging was performed in an operating room with a controlled temperature (18–22 °C) and humidity (50–55%). To reduce the temperature bias, thermal imaging was performed 10 min after the patient entered the operating room. The images were captured at a distance of 40 cm from the flaps. After monitoring, the dressing methods for all flaps were equally controlled with the use of ointments and gauze. If the location of the flap was in an area where splinting was possible, the instability of the flap was reduced through the use of a splint.

### 2.5. Infrared Thermal Image Analysis

**Figure 3:**
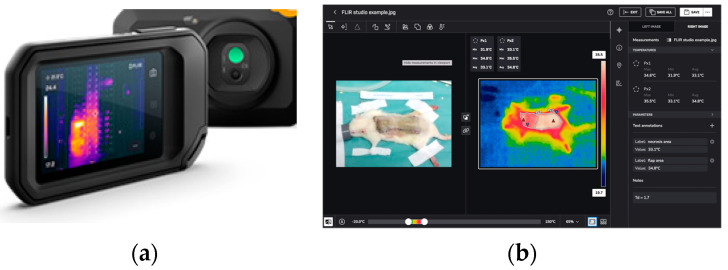
FLIR camera and thermal analysis software. (**a**) The FLIR C5 camera (Teledyne FLIR LLC, Wilsonville, OR, USA) has two cameras—a thermal imager (160 × 120 pixels) and 5-megapixel visual camera (640 × 480 pixels). (**b**) FLIR Thermal Studio software (Teledyne FLIR LLC, Wilsonville, OR, USA) is displayed on the right. It is used to analyze thermal images, which allows the user to identify the temperature of a specific point or area on the image and calculate the temperature difference.

The authors also used the FLIR Thermal Studio software 1.7 (Teledyne FLIR LLC, Wilsonville, OR, USA) to analyze the thermal images, which allows for the identification of the temperature of a specific point or area on a thermal image and calculate the temperature difference (Figure 3).

On the obtained thermal images, the authors marked the range of the total flap area and measured the average temperature value of the flap area (ATF) using an imaging software program. In addition, we measured the temperature of the perforator of the flap (PF), as well as the normal skin (NS) at the point 2 cm proximal from the flap in anatomical position. The temperature difference (dT) between normal skin and the perforator of the flap was compared. In addition, the temperature difference between the perforator and the average temperature of flap (AFP) was obtained.

### 2.6. Statistical Analysis

For the rat model study, paired *t*-tests were used to compare the mean temperature differences between the necrotic area and the total flap area at POD3 and POD7. Linear regression analysis assessed the relationship between pre-flap dorsal skin temperature and necrotic area temperature. Gaussian GLMMs were used to account for repeated measures within each rat, examining the association between necrotic area temperature and total flap temperature while considering individual variability.

Statistical significance was determined using the Mann–Whitney U test for independent groups. In addition to the Mann–Whitney U test, generalized linear mixed models (GLMM) were applied to analyze the temperature differences (NS-PF and PF-AFP) in relation to fixed effects such as sex, age, flap type, and flap necrosis. The models included random effects for each patient to account for intra-patient variability. The following formulae were used:

For NS-PF POD 7: NS-PF POD 7~Sex + Age + Flap type + Flap necrosis + (1|Patient)

For PF-AFP POD 7: PF-AFP POD 7~Sex + Age + Flap type + Flap necrosis + (1|Patient)

We also conducted *t*-tests to compare the temperature differences between pedicle flaps and free flaps in the clinical study.

Statistical analyses were performed using SPSS (ver 27; IBM Corp., Armonk, NY, USA) and R (version 4.0.3) with the “lme4” package. A *p*-value of less than 0.05 was considered statistically significant.

## 3. Results

### 3.1. Rat Models

**Figure 4:**
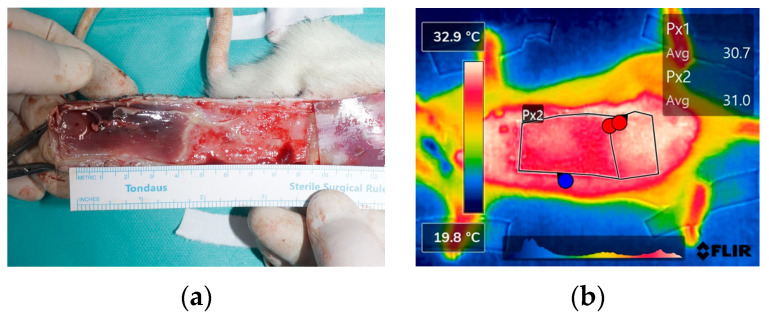
Analysis of flap necrosis in rat models. (**a**) After euthanization of the rats, the total flap and necrotic areas were measured with distinguishment of flap viability. (**b**) The thermal images were captured by FLIR camera, indicating the necrosis area (colored red), which shows a drop in temperature compared to the viable area (colored white). The temperature difference between the total flap area and necrotic area was calculated by using infrared thermal software.

The degree of necrosis of the flaps was evaluated by flap color and skin turgor on POD 3 and POD 7. The necrotic part of the flap was dark in color and appeared to dry out hard. On POD 7, all rats were euthanized, and sutures were removed to evaluate the undersurface of the flap, and the difference in necrosis of the flap was more noticeable on the undersurface of the flap. The mean surface area of the total flap was 27.12 ± 0.90 cm^2^, and the mean ratios of the total flap area over the necrotic area were 24.4% and 27.5% in POD 3 and POD7, respectively. On thermal imaging, the necrotic flap was clearly distinguished from the viable flap by color differences, with areas of high temperature being white and areas of low temperatures being red (Figure 4). The mean temperature difference in each area was 0.8°C in POD 3 and 0.792 °C in POD 7 (Table 1).

The paired *t*-test comparing the mean temperature differences between the necrotic area and the total flap area at POD3 and POD7 showed no significant difference (*t* = 0.084, *p* = 0.934). This indicates that the temperature difference between the necrotic area and the total flap area remains consistent over time(Table 2).

Linear regression analysis indicated that the pre-flap dorsal skin temperature did not significantly affect the necrotic area temperature at POD3 (*p* = 0.300) or POD7 (*p* = 0.315). This suggests that the initial dorsal skin temperature is not a significant predictor of the necrotic area temperature(Table 3).

The Gaussian GLMMs showed that the necrotic area temperature was significantly associated with the total flap temperature at both POD3 (*p* < 0.001) and POD7 (*p* < 0.001). However, the pre-flap temperature was not significantly associated with the necrotic area temperature at POD3 (*p* = 0.083) but was significant at POD7 (*p* = 0.003) (Table 4).

These findings suggest that the total flap temperature is a reliable predictor of the necrotic area temperature, supporting its use for early detection and monitoring of necrosis.

### 3.2. Patients

#### 3.2.1. Patient Demographics and Flap Characteristics

In total, 22 patients and 22 flaps were included in this study. There was a total of sixteen male and six female patients, with a total average age of 57.45 years (range: 27 to 100). All flaps were either pedicled flaps (fourteen in total) or free flaps (eight in total). Various pedicled flaps were made depending on the location of the defect and the surrounding vascularity, and an anterolateral thigh (ALT) free flap was used for free flap reconstruction in all cases except two. Necrosis occurred in two cases of the free flap group due to arterial insufficiency, and one case of total necrosis occurred in the free flap group due to venous congestion. In the pedicled flap group, one case of partial necrosis occurred. Initially, arterial insufficiency occurred in two cases, but despite salvage procedures being performed in two of these cases, flap failure occurred, and a new flap was reapplied. One of these cases survived stably without necrosis, so it was not included in the necrosis group. The location of the flap site varied from the scalp to the lower extremity. There was no other complication, such as hematoma or infection (Table 5 and Table 6).

#### 3.2.2. Infrared Thermal Imaging Analysis

The temperature difference between normal skin and the perforator (dT of NS-PF) in the non-necrosis group showed a gradual decrease over time, while that in the necrosis group increased.

In POD 0, the temperature difference between normal skin and perforator (dT of NS-PF) was shown to be lower in the necrosis group (1.55) than in the non-necrosis group (1.817). However, from POD 1 to 7, the necrosis group showed higher dT of NS-PF values than the non-necrosis group. Among them, there was a statistically significant difference between the non-necrosis group and the necrosis group in POD 3 and POD 7. When the necrosis group was subdivided, the difference between the total necrosis group and the non-necrosis group became higher. The difference between the perforator temperature and the average temperature of the flap (dT of PF-AFP) showed positive values from POD 0 to POD 7 in the non-necrosis group, while the necrosis group showed negative values from POD 1 to POD 7. On POD 1, the dT of PF-AFP was significantly different between the non-necrosis group (0.35) and the necrosis group (−0.175), with a *p*-value of 0.002. This trend continued with significant differences in POD 2 (*p* = 0.003), POD 3 (*p* < 0.001), and POD 7 (*p* < 0.001) (Table 7).

**Figure 5:**
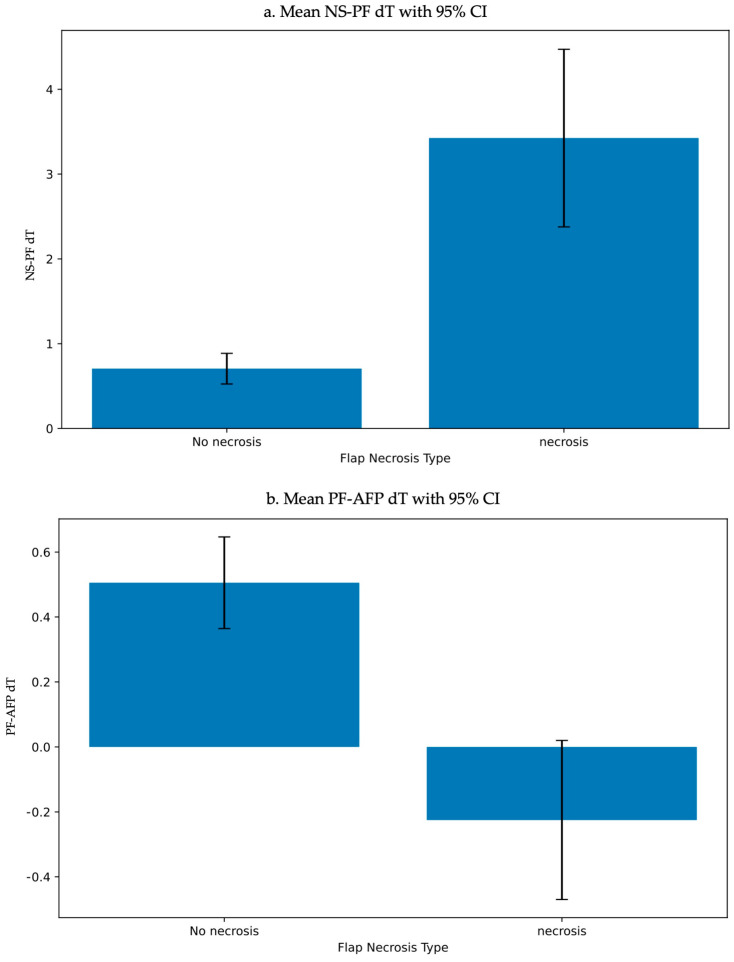
Mean temperature differences (NS−PF and PF−AFP) with 95% confidence intervals across different flap necrosis types. (**a**) Mean NS−PF temperature difference with 95% confidence intervals across different flap necrosis types. (**b**) Mean PF−AFP temperature difference with 95% confidence intervals across different flap necrosis types. Abbreviations: NS−PF, normal skin−perforator; PF−AFP, perforator−average of flap; CI, confidence intervals.

In addition to the infrared thermal imaging analysis, we applied generalized linear mixed models (GLMM) to analyze the temperature differences (NS-PF and PF-AFP) in relation to fixed effects such as sex, age, flap type, and flap necrosis. The models included random effects for each patient to account for intra-patient variability. The analysis revealed significant effects of flap necrosis on both NS-PF and PF-AFP temperature differences. Specifically, the necrosis group showed a significant impact on temperature differences, with a *p*-value < 0.001 in both models (Table 8 and Table 9). Figure 5 presents the mean temperature differences (NS-PF and PF-AFP) across different flap necrosis types, along with their 95% confidence intervals.

These results significantly contribute to understanding the impact of flap necrosis on NS-PF and PF-AFP temperature differences.

#### 3.2.3. Case of Non-Necrosis Group (Case #7 and Case #13)

**Figure 6:**
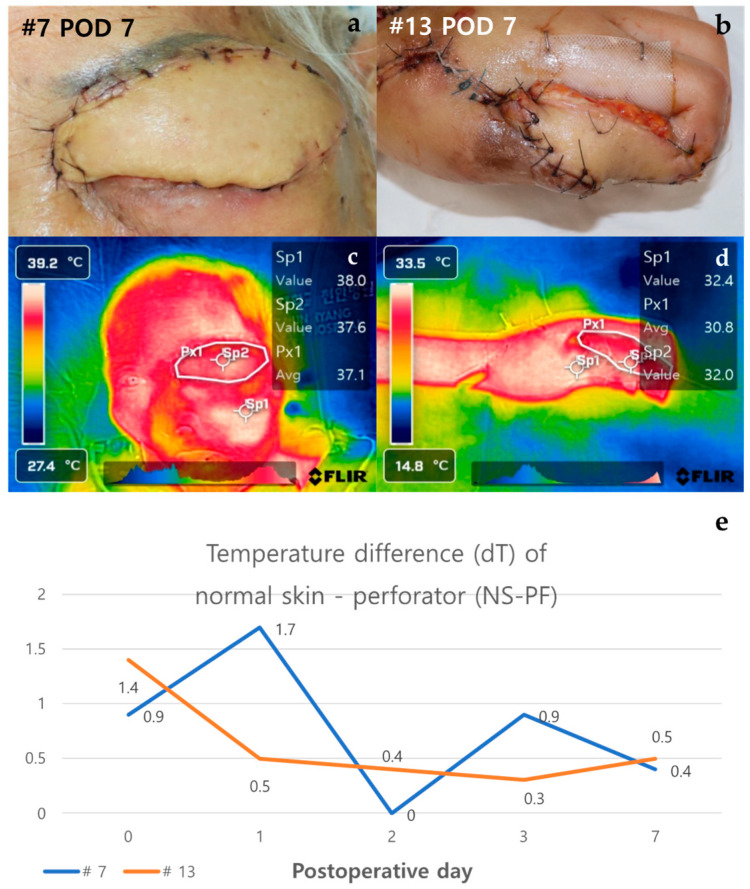
Analysis of cases of non-necrosis group (Case #7 and Case #13). (**a**) Case #7—A 100-year-old female patient underwent reconstruction through ALT free flap after tumor resection due to skin cancer on the left upper eyelid. On POD 7, the flap was maintained stably without any necrosis. (**b**) Case #13—A 23-year-old female patient who underwent SCIP free flap reconstruction after distal finger necrosis due to use of vasopressors. On POD 7, the flap has no necrosis. (**c**,**d**) In the thermal images of POD 7, both flaps have a similar color to the surrounding area except for some margins. (**e**) The graph shows NS-PF decreasing over time. Abbreviations: POD, postoperative day; dT, temperature difference; NS-PF, normal skin–perforator; ALT free flap, the anterolateral thigh free flap; SCIP free flap, the superficial circumflex iliac artery perforator free.

Case #7 is a 100-year-old female patient with no past medical history aside from squamous-cell carcinoma of the left upper eyelid. She underwent reconstruction through an ALT free flap after resection of the full-thickness skin, including the tumor on the left upper eyelid. Case #13, a healthy 23-year-old female patient, was reconstructed through superficial circumflex iliac artery perforator (SCIP) free flap after distal finger necrosis caused by the use of vasopressors due to septic shock. Both cases showed stable progress without necrosis, and the temperature difference between normal skin and perforator (dT of NS-PF) showed a decreasing trend over time (Figure 6). On POD 0, the dT of NS-PF for Case #7 and Case #13 were 0.9 and 1.4, respectively. However, by POD 7, the dT of NS-PF had decreased to 0.4 and 0.5 for Case #7 and Case #13, respectively, indicating a reduction in temperature difference over time. This decrease in dT of NS-PF reflects the stabilization and successful integration of the flap without vascular compromise. The thermal images of both cases on POD 7 showed a similar color to the surrounding area except for some margins, indicating no signs of necrosis. Both flaps survived stably without any necrosis, and the patients were discharged after POD 14.

#### 3.2.4. Case of Venous Congestion in Necrosis Group (Case #10 and Case #21)

**Figure 7:**
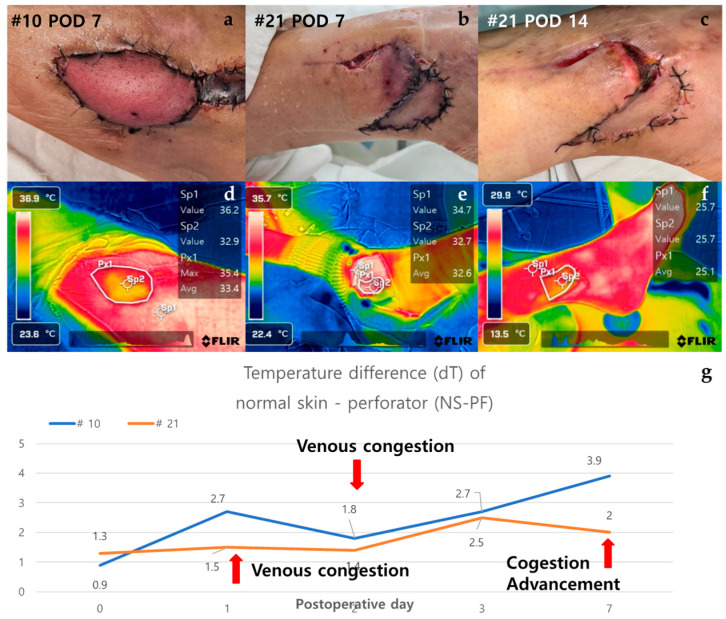
Analysis of cases of venous congestion in necrosis group (#10 and #21). Case #10 is a 51-year-old male patient who underwent ALT reconstruction for an ankle defect that had occurred after orthopedic surgery. The flap showed venous congestion since POD 1, and it gradually worsened. (**a**) On POD 7, the overall color of the flap was purple with edematous change. Case #21—A 49-year-old female patient underwent peroneal artery perforator-based flap for a defect that occurred after orthopedic surgery. Venous congestion in the flap started, with the flap color appearing purple since POD 2, and leech therapy for flap salvage was carried out. (**b**) On POD 7, the distal part of the flap still showed features of congestion, but the central part showed improvement with a lightening of color. (**c**) On POD 14, the necrotic area was demarcated, resulting in distal partial necrosis. (**d**,**e**) In the thermal images of POD 7, both flaps appeared yellow in color as the temperature dropped. (**f**) In the thermal image of Case #21 on POD 14, the difference in temperature decreased in comparison to POD7. (**g**) The graph showed an upward trend over time from the point of venous congestion, but in Case #21, the temperature difference decreased as the congestion improved. Abbreviations: POD, postoperative day; dT, temperature difference; NS-PF, normal skin–perforator; ALT, the anterolateral thigh.

Case #10 is a 51-year-old male patient who has been taking medication for diabetes for 15 years and underwent ALT free flap reconstruction for a defect in his left foot that occurred after orthopedic surgery. From POD 2, the color of the flap gradually turned purplish and showed a congestive appearance. Conservative salvage using heparin gauze was attempted, but the effect was not significant. Subsequently, venous congestion progressed, and the dT of NS-PF value continued to increase. By POD 7, the flap became darker in color and eventually led to total necrosis. The dT of NS-PF was 1.2 on POD 0 and increased to 3.5 by POD 7, indicating significant venous congestion and the resulting necrosis. Case #21 is a 49-year-old female patient with no medical history or medication history who suffered a right distal tibia–fibula fracture after a traffic accident. A peroneal artery perforator-based fasciocutaneous flap was performed to reconstruct a defect exposing the metal plate after reduction with plate fixation for an ankle fracture. From POD 1, the flap began to become hyperemic from the distal portion and progressed with a congestive appearance. Salvage was performed using leech therapy. Until POD 7, a similar appearance was maintained, but thereafter, the central portion showed improvement in congestion, and only partial necrosis of the distal portion remained on POD 14. The dT of NS-PF initially showed an increasing trend, being 1.0 on POD 0 and 2.5 on POD 7, but later decreased to 0 by POD 14, indicating improvement in congestion and partial recovery (Figure 7).

#### 3.2.5. Case in which a New Flap was Applied after Salvage Procedure for Arterial Insufficiency (Case #12 and Case #14)

**Figure 8:**
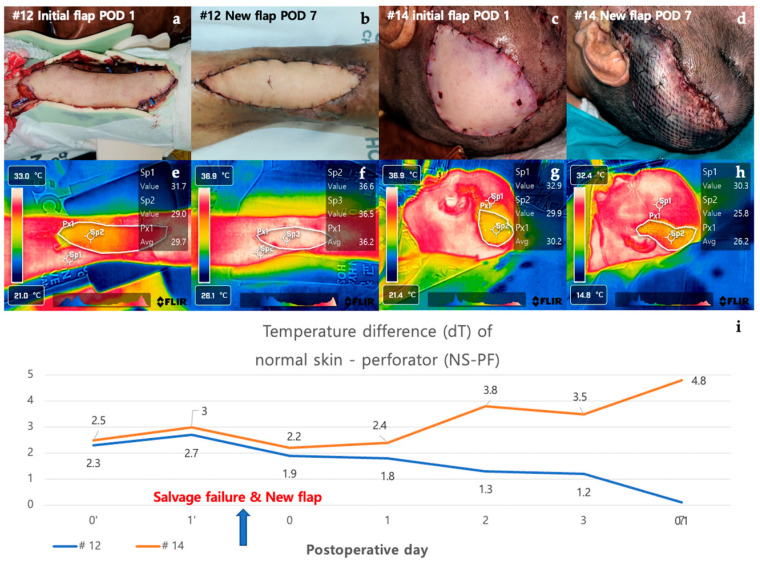
Analysis of cases in which a new flap was applied after salvage procedure for arterial insufficiency (#12 and #14). Case #12 is a 48-year-old male patient who underwent TDAP free flap for a defect that occurred after orthopedic surgery due to a fracture of the left distal tibia. (**a**) On POD 1, the color of the flap was pale and mottled, indicating arterial occlusion of the flap. The salvage procedure, re-anastomosis, failed and was covered with a new ALT free flap. (**b**) The new flap remained stable without necrosis on POD 7. Case #14 is a 75-year-old male patient who underwent ALT free flap for a defect caused by a surgical site infection of the scalp. (**c**) As with Case #12 on POD 1, the color of the flap was pale and mottled, indicating arterial occlusion of the flap. The salvage procedure in the form of a re-anastomosis failed and was covered with a new vastus lateralis muscle free flap. (**d**) The flap showed signs of arterial occlusion even after reoperation, and it progressed to total necrosis with overall darkening of color. (**e**,**f**) When there was arterial occlusion, the flap appeared yellow overall, but when the new flap remained stable, it appeared white. (**g**,**h**) The flap had an overall yellow color on thermal imaging due to arterial occlusion. (**i**) The two graphs showed a pattern of increasing temperature difference as arterial occlusion occurred in the early stages but showed a contrasting course after the application of a new flap. Abbreviations: POD, postoperative day; dT, temperature difference; NS-PF, normal skin–perforator; TDAP free flap, the thoracodorsal artery perforator free flap; ALT free flap, the anterolateral thigh free flap.

Case #12 is a 48-year-old male patient who underwent a thoracodorsal artery perforator (TDAP) free flap for a defect that occurred after orthopedic surgery due to a fracture of the left distal tibia. On POD 1, the Doppler sound of the anastomosed vessel could not be heard, and it was judged as arterial occlusion. Despite the salvage procedure, the flap failed, and an ALT free flap was reapplied. The new flap remained stable without necrosis on POD 7. Initially, the dT of NS-PF was 2.0 on POD 0 but increased to 2.8 on POD 1, reflecting the arterial insufficiency. After the application of the new flap, the dT of NS-PF decreased to 0.7 on POD 7, indicating the success of the new flap. Case #14 is a 75-year-old male patient who underwent burr-hole drainage several times due to subdural hemorrhage and was hospitalized due to surgical site infection. ALT free flap was performed for a scalp defect where a plate fixed to the skull after the site of the craniotomy was exposed. On POD 1, the flap was pale in the center and mottled around the margins of the flap, suggesting arterial occlusion. Despite the salvage procedure, the flap failed, and a vastus lateralis muscle free flap was performed. However, the new flap also showed signs of arterial occlusion and progressed to total necrosis. Initially, the dT of NS-PF was 1.8 on POD 0 and increased to 3.0 on POD 1, similar to Case #12. However, after the new flap was applied, the dT of NS-PF further increased to 3.5 on POD 7, indicating continued vascular compromise and eventual total necrosis. The contrasting clinical courses of the two cases were clearly distinguished by the decrease and increase in the dT of NS-PF values. In Case #12, the successful salvage with a new flap was reflected by a significant decrease in dT of NS-PF, while in Case #14, the failure of the new flap was indicated by the continued increase in dT of NS-PF. (Figure 8).

#### 3.2.6. The Difference between the Temperature of Perforator Area and the Average Temperature of Total Flap (dT of PF-AFP)

On POD 0, both the necrosis and non-necrotic groups showed positive PF-AFP values, with 0.539 in the non-necrosis group and 0.175 in the necrosis group, showing no significant difference (*p* = 0.118). However, from POD 1 to POD 7, the necrosis group showed positive values, while the non-necrosis group showed negative values. This difference was statistically significant (Table 7).

#### 3.2.7. Comparison of Pedicle Flaps and Free Flaps

We performed statistical analyses to compare the temperature patterns between pedicled flaps and free flaps. Temperature differences were analyzed using paired *t*-tests across various postoperative days (PODs). The results indicate that there were no statistically significant differences in the temperature patterns between the two flap types at any time point (*p* > 0.05 for all comparisons) (Table 10).

## 4. Discussion

Skin grafts and flap surgeries are fundamental in plastic surgery for defect closure when primary or secondary intention is not feasible. Skin grafts are suitable for superficial defects with good vascular beds, whereas flap surgeries are vital for areas requiring more complex reconstructions involving full-thickness tissue. Among flap surgeries, pedicled flaps retain their original blood supply and are used for shorter distances between donor and recipient sites, offering reliability and reduced risk of vascular complications. Free flaps involve microsurgical reconnection of blood vessels at the recipient site, allowing for complex reconstructions over longer distances [8,9].

Flap surgery remains prevalent due to the increasing incidences of malignant tumors and the need for immediate breast reconstruction following mastectomy [10,11]. The most critical factor affecting flap survival is vascular compromise, primarily arterial occlusion and venous congestion [12,13]. The pathophysiology of arterial occlusion involves insufficient oxygen delivery and harmful metabolite accumulation, leading to inflammation and tissue necrosis. Venous congestion results in increased intravascular pressure, causing edema and further tissue damage [14,15]. Therefore, immediate and accurate postoperative flap monitoring is crucial to ensure flap survival and prevent complications.

In reconstructive microsurgery, perforator flaps have become significant due to their ability to preserve major vessels and muscle function while allowing less invasive reconstruction. They enable complex three-dimensional reconstructions with improved outcomes and reduced donor site morbidity [16]. Conventional flap monitoring relies on clinical observation of skin color, turgor, temperature, and capillary refill time, which can be subjective and dependent on the surgeon’s experience [2,17]. Specific signs indicate whether blood flow issues are due to arterial insufficiency or venous congestion. Arterial insufficiency causes the flap to appear pale or white, feel cool, and have a prolonged capillary refill time, often leading to necrosis if untreated. Venous congestion presents with cyanosis (a blue or dusky appearance), swelling, and a delayed capillary refill time, with the flap feeling warm despite the congestion [13]. Besides these subjective methods, various objective devices are used for flap monitoring [17,18,19,20,21,22,23] (Table 11). However, no universally established gold standard exists among these modalities. Each method has limitations, such as the need for specialized equipment, potential invasiveness, and operator dependency.

Various studies related to infrared thermography for flaps have been conducted for a long time, and interest in this field has recently increased. Many studies, such as burn depth analysis and preoperative perforator mapping, have been conducted, and there are also many existing studies that apply infrared thermography to flap monitoring [24,25]. These studies show a diverse range of methods and results.

In our rat model study, we used a caudally based reverse McFarlane flap model in rats to intentionally induce flap necrosis. This model effectively studies necrosis due to the progressive decrease in vascularity in the distal flap portion over time, leading to necrosis [26,27]. Understanding the hemodynamics of anterograde and reverse flaps is crucial. Anterograde flaps have blood flowing naturally from the body center toward the periphery, resulting in stable hemodynamics and higher survival rates. In contrast, reverse flaps have blood flow in the opposite direction, increasing vascular resistance and making the flap more prone to ischemia and necrosis [28,29,30,31].

Thermal imaging monitored flap changes, visualizing temperatures in color gradients (white to blue for decreasing temperatures). Necrotic areas consistently showed lower temperatures compared to viable tissue, particularly on the flap’s undersurface. At POD 3 and POD 7, there were no significant temperature differences (Table 1), likely due to the influence of the rats’ core body temperature, which may have slightly underestimated the gradient between necrotic and non-necrotic areas [32]. This suggests internal physiological factors help maintain temperature stability.

The pre-flap dorsal skin temperature was not significantly correlated with necrotic area temperature (Table 3), indicating the initial skin condition before surgery did not significantly affect temperature outcomes in necrotic regions. However, necrotic areas consistently exhibited lower temperatures compared to non-necrotic areas at both POD 3 and POD 7 (Table 4). This consistent finding, regardless of core body temperature or external factors, supports using necrotic area temperature as a reliable marker for early detection and monitoring of necrosis. Identifying necrotic areas through temperature differences underscores thermal imaging’s utility as a non-invasive and effective tool for assessing flap viability and ensuring timely intervention. These results highlight the importance of considering internal and external factors when evaluating flap necrosis and demonstrate thermal imaging’s robust applicability for clinical use in early detection and continuous monitoring of necrotic areas.

In the clinical cases, this study obtained the data by comparing the temperature between normal skin and the perforator of the flap and comparing the temperature of the perforator point of the flap with the average temperature of the entire flap area using infrared thermal imaging in the patients’ group.

**Figure 9:**
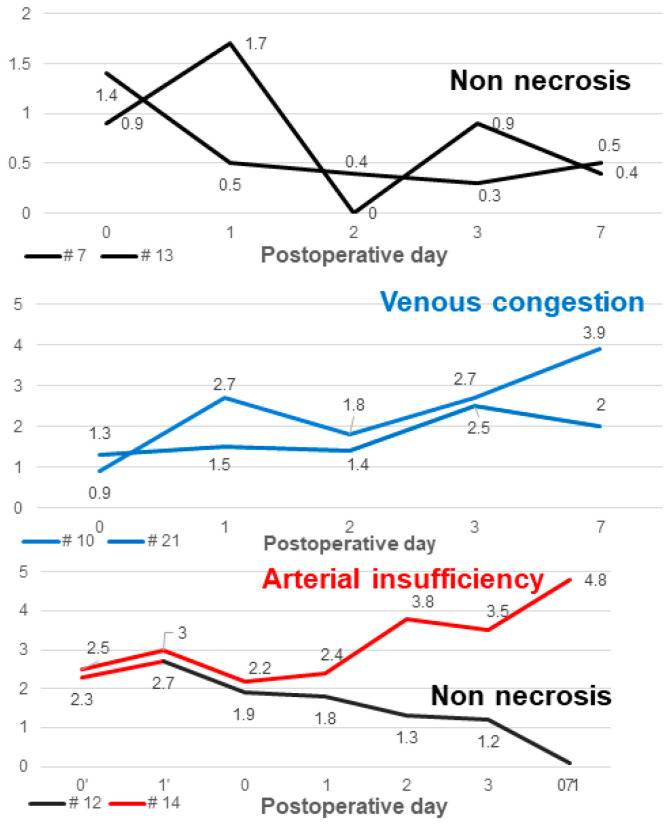
The graph of the temperature difference between normal skin and perforator according to clinical course of the flaps. The black graph, which represents the non-necrosis group, shows a downward trend in values over time. The red graph represents the group with arterial insufficiency, and the blue graph represents the group with venous congestion. Both red and blue show an upward trend in values over time.

In R. Kraemer’s study, they evaluated flap skin temperature and capillary microcirculation through postoperative monitoring using a digital infrared surface thermometer combined with laser Doppler and photospectrometry [33]. They proved their hypothesis that the flap skin temperature decreases when there is arterial thrombosis or venous compromise. The other previous studies demonstrated that if microvascular compromise occurs, the flap surface temperature decreases, and the temperature difference with adjacent skin increases [34,35]. In the same context as these, the non-necrosis group showed a decreasing trend in the temperature difference between normal skin and perforator (dT of NS-PF) over time, but cases with vascular compromise showed an upward trend in the temperature difference between normal skin and flap due to the flap’s temperature dropping further (Figure 9). Although this study did show a statistically significant difference in POD 3 and POD 7, there was no statistically significant difference between the non-necrosis group and the necrosis group until POD 2. However, because early rapid detection of vascular compromise and salvage of the flap is important, it should not be considered absolute guidance but can be used as a supplementary indicator [2].

In addition to the infrared thermal imaging analysis, we applied generalized linear mixed models (GLMM) to analyze the temperature differences (NS-PF and PF-AFP) in relation to fixed effects such as sex, age, flap type, and flap necrosis. The models included random effects for each patient to account for intra-patient variability. The GLMM analysis revealed significant effects of flap necrosis on both NS-PF and PF-AFP temperature differences, with a *p*-value < 0.001 in both models. Specifically, flap necrosis showed a significant increase in temperature difference in the necrosis group compared to the non-necrosis group, underscoring the potential of using these temperature differences as reliable indicators for early detection and monitoring of flap necrosis.

When comparing temperature patterns between pedicled and free flaps, statistical analyses revealed no significant differences. Temperature differences were analyzed using paired *t*-tests across various postoperative days (PODs). The results indicate that there were no statistically significant differences in the temperature patterns between the two flap types at any time point (*p* > 0.05 for all comparisons). This finding suggests that both flap types can be monitored similarly using infrared thermal imaging.

In Cases #7 and #13, the dT of NS-PF value decreased as time passed compared to the initial stage, but in Cases #10 and #21, which showed venous congestion, the temperature of the perforator decreased compared to normal skin as time passed, and the dT of NS-PF value increased. However, in Case #21, the temperature of the perforator increased again from POD 7 as congestion improved through leech therapy, and it ultimately progressed to partial necrosis rather than total necrosis. Therefore, when venous congestion occurs, it indicates that if the dT of NS-PF value does not increase steadily but decreases, the progression to total necrosis may not occur (Figure 7).

Case #12 and Case #14 are cases in which salvage procedures and new flap applications were performed on POD 1 due to arterial occlusion. In both cases, the dT of NS-PF value on POD 1 was higher than the average of the non-necrosis group. In addition, when comparing Case #14 with Case #10, where venous congestion is represented, it can be confirmed that the dT of NS-PF value is higher in Case #14 with arterial insufficiency than in Case #10 with venous congestion. However, after applying a new flap, a contrasting, downward trend of dT of NS-PF value can be confirmed depending on the presence or absence of flap necrosis (Figure 8).

**Figure 10:**
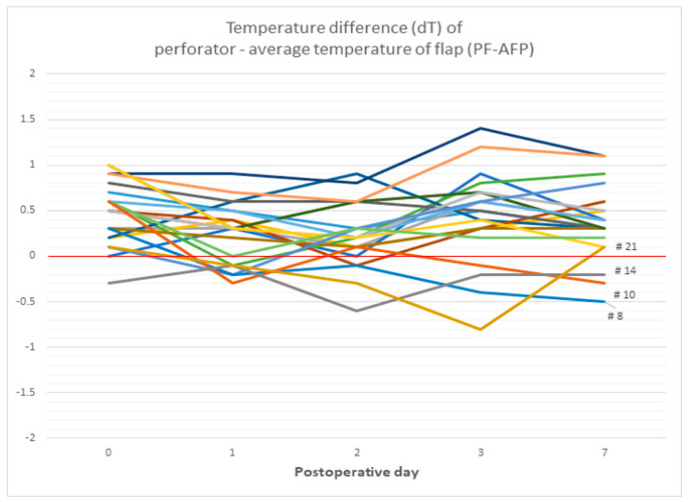
The graph of the temperature difference between perforator and mean temperature of flap. The temperature difference between perforator and average of flap (dT of PF-AFP) in the non-necrosis group showed a positive value in all cases from POD 0 to POD 7, except for 1–2 points, while in the necrosis group, it almost always showed a negative value.

In Figure 9, the temperature difference between perforator and average of flap (dT of PF-AFP) in the non-necrosis group showed a positive value in all cases from POD 0 to POD 7, except for 1–2 points, while in the necrosis group, it almost always showed a negative value (Figure 10). A positive value of dT of PF-AFP can be interpreted as the temperature of the perforator being higher than the average temperature of the flap, with a negative value meaning the opposite. Whitaker IS et al. used a dynamic infrared thermal camera for mapping the deep inferior epigastric artery perforator by performing a cold challenge, utilizing the hot spot appearing on the camera to measure the temperature at the perforator [36]. Perng CK et al. explained that the flap’s surface temperature is influenced by heat from blood flow, heat conduction from underlying tissue, and heat loss to the air [37]. Applying these concepts to our study, comparing the average temperature of the perforator and the flap allows us to unify other heat sources and focus on blood flow. Since all flaps were imaged after being at a lower room temperature, this can be seen as a cold challenge. While the perforator’s temperature might not be visually distinct from the flap, thermal imaging analysis software allows for precise comparison. If the dT of PF-AFP is less than 0, it suggests microvascular compromise in the flap.

Many previous studies have analyzed temperature using two methods in flap monitoring through infrared thermography. The first was to measure the absolute temperature gradient of the flap itself, and the other was to measure the gradient in temperature difference between the flap and adjacent normal skin. This study utilized the latter method but showed limitations in early flap monitoring due to variables in the temperature measurement of normal skin. Since comparisons cannot be made between flaps on different patients or on different sites, there is a lot of bias toward environmental variables. However, comparing perforator temperature and average temperature within the flap itself can reduce other biases and allow a more accurate evaluation of vascular compromise.

There are several limitations to this study. First, the sample size was too small to effectively evaluate the efficacy of flap monitoring through thermal imaging. The small number of necrosis cases and inconsistent flap sizes/types likely prevented statistical significance. Second, we measured the temperature of normal skin at a specific point, which may not represent overall skin temperature accurately. S. Hummelink et al. used the mean temperature within a specific area for comparison instead [34]. Additionally, the FLIR camera can be influenced by environmental factors, introducing biases. MA Moran-Romero et al. noted that thermal imaging for free flap monitoring could produce ambiguous results [38]. To mitigate this, we conducted measurements in a controlled surgical theater environment and analyzed the gradient of temperature differences rather than absolute values.

Evaluating microvascular flow using absolute infrared thermal values is challenging. However, the gradient of temperature differences between normal skin and the perforator over time can guide the interpretation of flap health. This study proposes a novel method for flap monitoring using the temperature difference between the perforator and the average flap temperature as a supplementary indicator of vascular compromise. The FLIR camera’s non-contact nature and convenience make it a valuable tool for flap monitoring.

## 5. Conclusions

Numerous studies are investigating flap monitoring using infrared thermal imaging. FLIR cameras offer the benefits of being non-invasive and highly convenient. While further research is necessary before FLIR cameras can serve as definitive indicators for vascular compromise during flap monitoring, they can effectively function as auxiliary tools for assessing and predicting the overall clinical progression of flaps. This study introduces a novel method for flap monitoring via infrared thermography by analyzing the temperature differential between the perforator area and the average flap temperature, demonstrating significant potential for enhancing the flap monitoring process.

## Figures and Tables

**Table 1:** Thermal analysis of rat models.

Flap Models (*n* = 28)	POD 3	POD 7
Necrotic area/total flap ratio (%)	24.4	27.5
Temperature difference between total flap and necrotic area	0.800	0.792

Abbreviations: POD, postoperative day.

**Table 2:** Summary of paired *t*-test results.

Statistic	Value
*t*-value	0.084
*p*-value	0.934

**Table 3:** Summary of regression analysis results.

Dependent Variable	Coefficient	Std. Error	*t*-Value	*p*-Value
POD3 Necrotic Area Temp				
Intercept	28.069	4.663	6.020	0.000
Before Flap Temp	0.149	0.141	1.057	0.300
POD7 Necrotic Area Temp				
Intercept	27.592	5.045	5.469	0.000
Before Flap Temp	0.156	0.153	1.024	0.315

Abbreviations: Std. error, standard error; POD, postoperative day; Temp, temperature.

**Table 4:** Summary of GLMM results.

Variable	Coefficient	Std. Error	z-Value	*p*-Value	95% CI Lower	95% CI Upper
Intercept (POD3)	0.274	1.126	0.243	0.808	−1.934	2.481
Before Flap Temp	−0.040	0.023	−1.734	0.083	−0.085	0.005
POD3 Total Flap Temp	1.007	0.031	32.568	0.000	0.946	1.068
Intercept (POD7)	0.003	0.881	0.003	0.997	−1.724	1.730
Before Flap Temp	−0.004	0.001	−2.988	0.003	−0.006	−0.001
POD7 Total Flap Temp	0.980	0.025	39.099	0.000	0.931	1.029

Abbreviations: GLMN, generalized linear mixed models; Std. error, standard error; CI, confidence interval; POD, postoperative day; Temp, temperature.

**Table 5:** Patients demographics.

No. of Patient	Sex	Age	Location	Flap	Flap Type	Flap Necrosis
1	M	61	Lt. ankle	ALT free flap	Free flap	No necrosis
2	F	27	Scalp	ALT free flap	Free flap	No necrosis
3	M	16	Lt. ankle	ALT free flap	Free flap	No necrosis
4	M	55	Lt. lower leg	ALT free flap	Free flap	No necrosis
5	M	72	Philtrum	ALT free flap	Free flap	No necrosis
6	F	66	Lt. ankle	ALT free flap	Free flap	No necrosis
7	F	100	Lt. upper eyelid	ALT free flap	Free flap	No necrosis
8	M	60	Lt. foot	ALT free flap	Free flap	Total necrosis
9	M	63	Lt. ankle	ALT free flap	Free flap	No necrosis
10	M	51	Lt. foot	ALT free flap	Free flap	Total necrosis
11	F	70	Rt. foot	ALT free flap	Free flap	No necrosis
12	M	48	Lt. ankle	TDAP free flap → ALT free flap	Free flap	No necrosis
13	F	23	Lt. hand	SCIP free flap	Free flap	No necrosis
14	M	75	Scalp	ALT free flap → vastus lateralis muscle free flap	Free flap	Total necrosis
15	M	81	Nose	Nasolabial fold flap	Pedicled flap	No necrosis
16	M	56	Nose	Nasolabial fold flap	Pedicled flap	No necrosis
17	M	52	Nose	Nasolabial fold flap	Pedicled flap	No necrosis
18	M	91	Nose	Paramedian forehead flap	Pedicled flap	No necrosis
19	M	57	Nose	Paramedian forehead flap	Pedicled flap	No necrosis
20	M	57	Rt. lower leg	ALT pedicled flap	Pedicled flap	No necrosis
21	F	49	Rt. ankle	Peroneal artery perforator-based FC rotation flap	Pedicled flap	Partial necrosis

Abbreviations: Rt, right; Lt, left; ALT, the anterolateral thigh; TDAP, the thoracodorsal artery perforator; SCIP, the superficial circumflex iliac artery perforator; FC, fasciocutaneous.

**Table 6:** Flap characteristics.

	Necrosis Group (n = 4)
Necrosis type	
Total necrosis	3
Partial necrosis	1
Vascular compromise	
Arterial insufficiency	2
Venous congestion	2

**Table 7:** Analysis of comparison between non-necrosis group and necrosis group.

	Non-Necrosis (n = 18)	Necrosis (n = 4)	Total Necrosis (n = 3)	Partial Necrosis (n = 1)
Age (years)	57.167	58.75		
Sex				
Male	13	3		
Female	5	1		
Temperature difference (dT) between normal skin and perforator (NS-PF) (°C)				
POD 0	1.817	1.55 (0.484)	1.633 (0.740)	1.3 (0.526)
POD 1	1.533	1.875 (0.434)	2 (0.262)	1.5 (0.842)
POD 2	1.528	2.2 (0.434)	2.467 (0.262)	1.4 (0.842)
POD 3	0.989	2.525 (0.001 *)	2.533 (0.006 *)	2.5 (0.105)
POD 7	0.706	3.5 (<0.001 *)	4 (0.002 *)	2 (0.105)
Temperature difference (dT) between perforator and average of flap (PF-AFP) (°C)				
POD 0	0.539	0.175 (0.118)	0.2	0.1
POD 1	0.35	−0.175 (0.002 *)	−0.2	−0.1
POD 2	0.333	−0.225 (0.003 *)	−0.2	−0.3
POD 3	0.617	−0.375 (<0.001 *)	−0.233	−0.8
POD 7	0.489	−0.225 (<0.001 *)	−0.333	−0.1

Note: Values are expressed as means. The value between the parentheses corresponds to the *p*-value for statistical significance between the group and the non-necrosis group. * *p*-value < 0.05. Abbreviations: POD, postoperative day; dT, temperature difference; NS-PF, normal skin–perforator; PF-AFP, perforator–average of flap.

**Table 8:** NS-PF POD 7 model results.

Variable	Coefficient	*p*-Value
Sex (male)	0.475	<0.001
Flap type (free flap)	0.185	0.611
Flap type (pedicled flap)	−0.647	0.003
Flap necrosis (necrosis)	2.631	<0.001

Abbreviations: NS-PF, normal skin–perforator.

**Table 9:** PF-AFP POD 7 model results.

Variable	Coefficient	*p*-Value
Sex (male)	−0.258	0.088
Flap type (free flap)	−0.070	0.768
Flap type (pedicled flap)	0.093	0.515
Flap necrosis (necrosis)	−0.716	<0.001

Abbreviations: PF-AFP, perforator–average of flap.

**Table 10:** T-test results for temperature differences between pedicled and free flaps.

POD	*t*-Statistic	*p*-Value
NS-PF POD 0	0.927	0.366
NS-PF POD 1	0.271	0.789
NS-PF POD 2	−0.217	0.831
NS-PF POD 3	−0.656	0.520
NS-PF POD 7	−1.547	0.139
PF-AFP POD 0	1.554	0.138
PF-AFP POD 1	−0.054	0.957
PF-AFP POD 2	0.326	0.748
PF-AFP POD 3	0.068	0.946
PF-AFP POD 7	0.610	0.550

Abbreviations: POD, postoperative day; NS-PF, normal skin–perforator; PF-AFP, perforator–average of flap.

**Table 11:** Modalities used in flap monitoring.

Monitoring Method	Advantages	Limitations	References
Clinical Examination	Non-invasive Widely available Low cost	Limited applicability in buried flaps Risk of poor interrater agreement due to inconsistent flap (failure) appearances	[18]
Acoustic Doppler Sonography	Non-invasive High sensitivity and specificity Real-time monitoring	Limited applicability in buried flaps Operator-dependent Limited ability to detect venous thrombosis	[17,18]
Implantable Doppler	Continuous monitoring High sensitivity and specificity Real-time monitoring	Invasive Requires surgical implantation Risk of infection Limited applicability in buried flaps	[17,18]
Indocyanine Green Fluorescence Angiography	Non-invasive High sensitivity and specificity Real-time monitoring Ability to detect venous thrombosis	Limited applicability in buried flaps Requires specialized equipment Limited ability to detect arterial thrombosis	[18,20]
Near-Infrared Spectroscopy	Non-invasive Real-time monitoring Ability to detect arterial thrombosis	Limited applicability in buried flaps Requires specialized equipment Limited ability to detect venous thrombosis	[19]
Tissue Oximetry	Non-invasive Real-time monitoring Ability to detect arterial thrombosis	Limited applicability in buried flaps Requires specialized equipment Limited ability to detect venous thrombosis	[12]
Transcutaneous Oximetry Measurement	Non-invasive Quantifying measurement Potential for thermal injury	Limited applicability in buried flaps Time required for measurement Low sensitivity	[23]

## Data Availability

The original contributions presented in the study are included in the article, further inquiries can be directed to the corresponding author.
